# Supplementary material for: Lack of RAC1 in macrophages protects against atherosclerosis
Source: PLoS One. 2020 Sep 17;15(9):e0239284. doi: 10.1371/journal.pone.0239284 (PMC7498073; doi:10.1371/journal.pone.0239284)
Supplement: S1 File — (PDF) [file pone.0239284.s001.pdf]

Supporting information

For detailed information, please see Material and Methods.

Figure legends

S1 Fig. Unaltered expression of RAC1 in SMCs in human advanced atherosclerotic carotid arteries. Number of SMCs expressing RAC1 within the intimal thickening of both intermediate and advanced atherosclerotic plaques. Mean ± SEM values (n = 9 in each). Wilcoxon signed rank test was used. p=0.09.

S2 Fig. Deficiency of RAC1 alters neither macrophage proliferation nor migration. (**A**) Proliferated number of $Rac1_{fl/fl}$ and $Rac1_{fl/fl}/LC$ BMMs up to 5 days. (**B**) Migrated number of $Rac1_{fl/fl}$ and $Rac1_{fl/fl}/LC$ BMMs at 4 and 16 hours. Mean ± SEM values of at least triplicated data. Student *t*-test was used. p>0.05.

S3 Fig. Unaltered intimal collagen composition in aortic arches deficient for RAC1 in macrophages. (**A**) Representative images of aortic arch sections stained with Sirius red. Arrowhead points to the internal elastic lamina bordering the intimal thickening from the medial layer. Scale bars represent 100 µm. (**B**) Areas of intimal formation were analyzed in $Rac1_{fl/fl}$ (n = 9) and $Rac1_{fl/fl}/LC$ (n = 11). Percentage of red color positivity was normalized to $Rac1_{fl/fl}$. p>0.05.

S4 Fig. Increased secretion of triglyceride in mice deficient for RAC1 in macrophages. Blood levels of cholesterol (A) and triglyceride (B) in fast protein liquid chromatography–fractionated plasma pooled from Rac1fl/fl mice (n = 5) that were infected with AdPCSK9 as compared to Rac1fl/fl/LC mice (n = 5) that were infected with AdPCSK9 after a high-fat diet for 24 weeks.

S5 Fig. Uncropped images of agarose gels and immunoblots containing bands represented in figures. The origin of DNA amplicons and protein bands that were cut and used in Figures 2A, 2B, 5D 6B and 6C have been highlighted in rectangular red frames.

S1 Fig

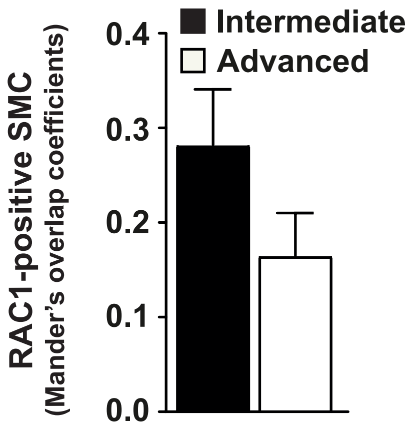

RAC1-positive SMC
(Mander's overlap coefficients)
0.4
0.3
0.2
0.1
0.0
Intermediate
Advanced

S2 Fig

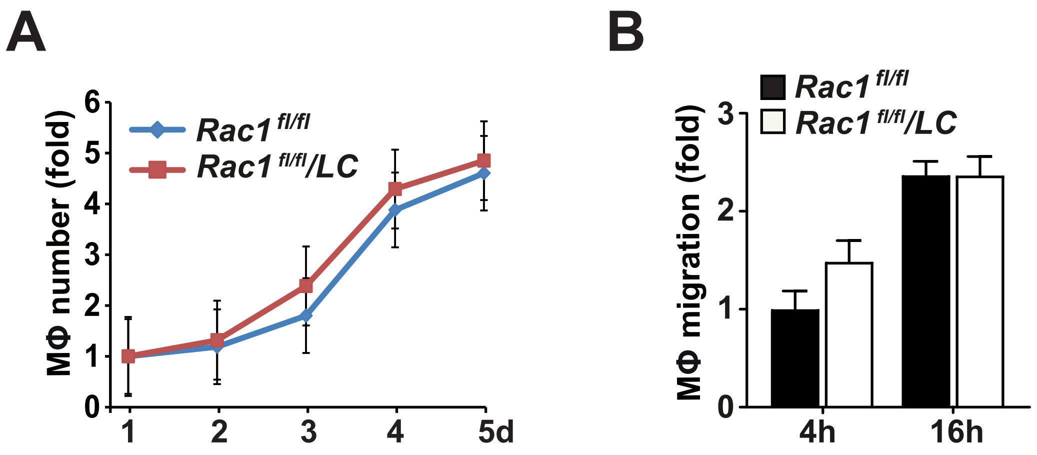
A
B
MΦ number (fold)
Rac1 fl/fl
Rac1 fl/fl/LC
0
1
2
3
4
5
6
1
2
3
4
5d
MΦ migration (fold)
0
1
2
3
4h
16h

S3 Fig

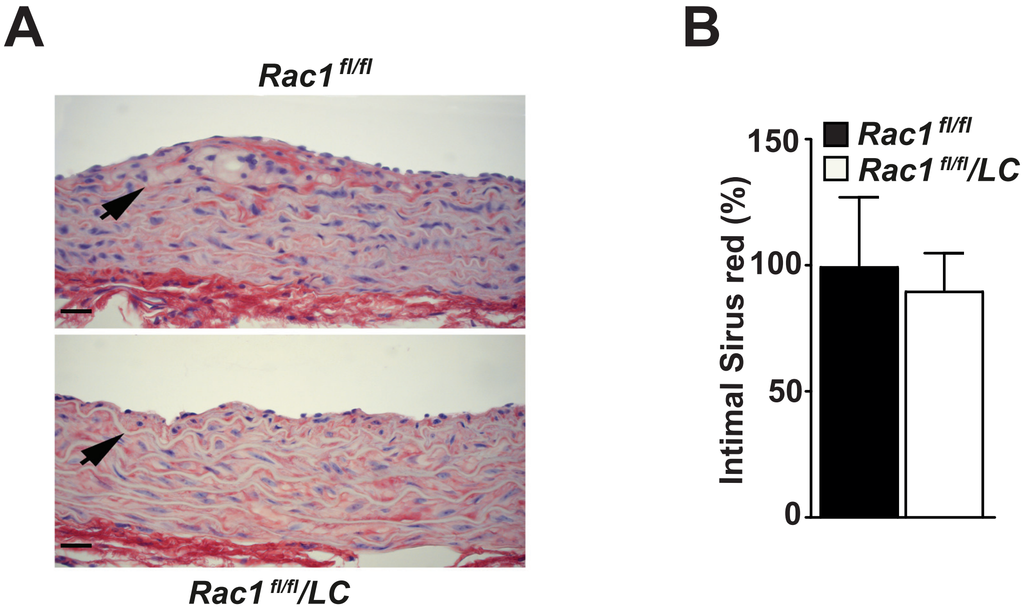

A
Rac1 fl/fl
Rac1 fl/fl/LC
B
Rac1 fl/fl
Rac1 fl/fl/LC
Intimal Sirus red (%)
150
100
50
0

S4 Fig

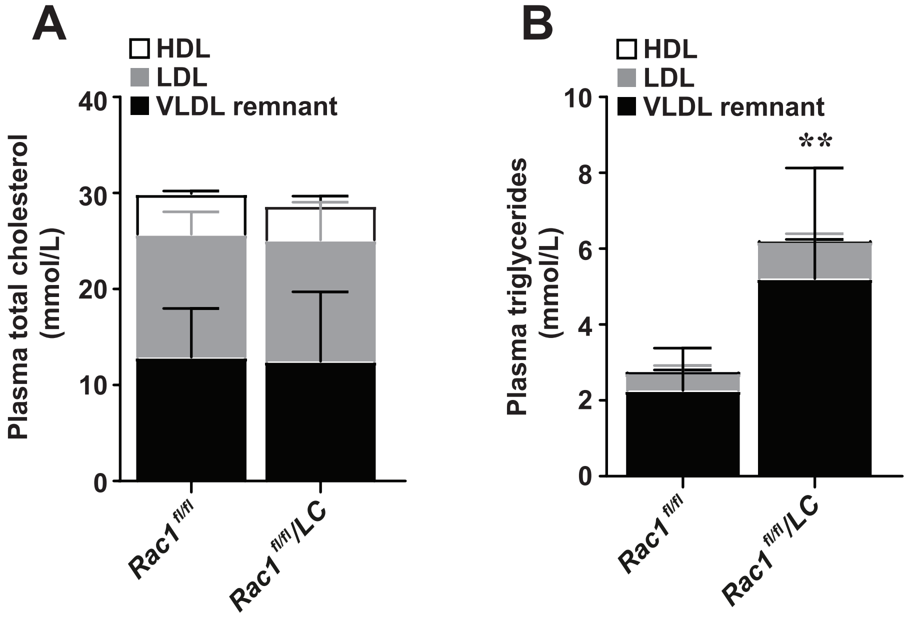

A
HDL
LDL
VLDL remnant
Plasma total cholesterol (mmol/L)
0
10
20
30
40
Rac1 fl/fl
Rac1 fl/fl/LC
B
HDL
LDL
VLDL remnant
Plasma triglycerides (mmol/L)
0
2
4
6
8
10
**
Rac1 fl/fl
Rac1 fl/fl/LC

S4 Fig

Uncropped immunoblots

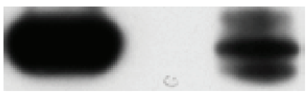

Figure 6B

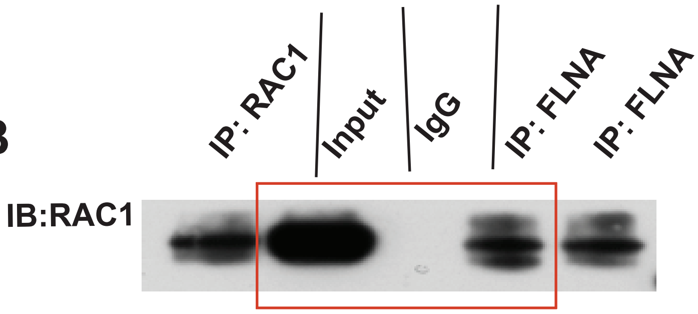

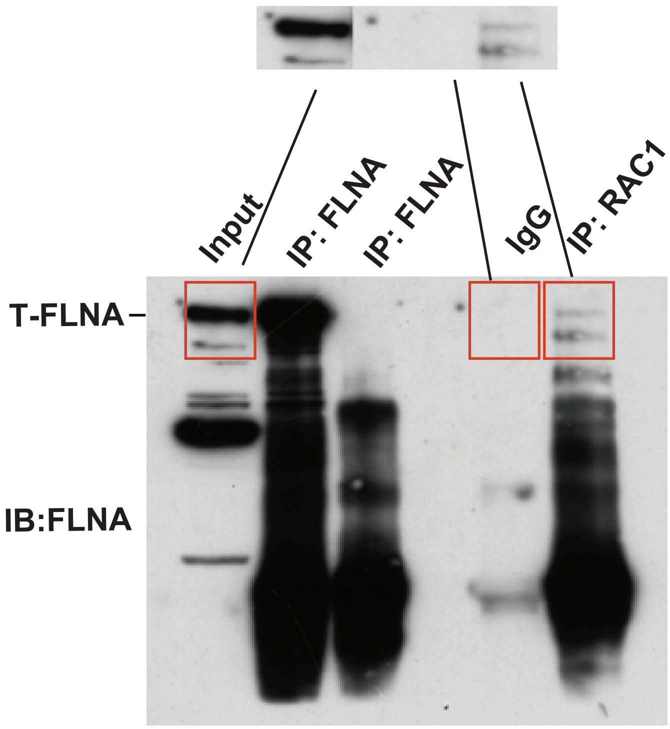

Figure 6C

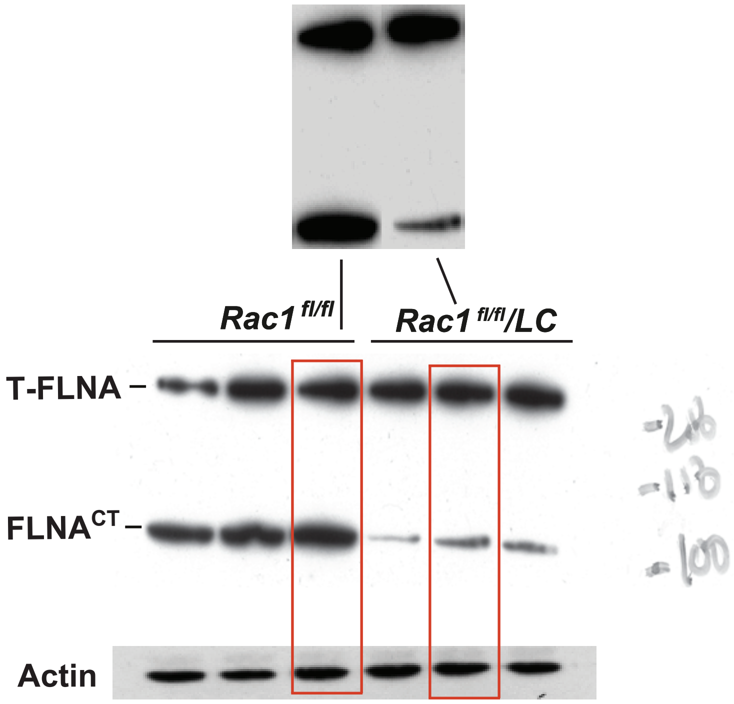

Figure 2A

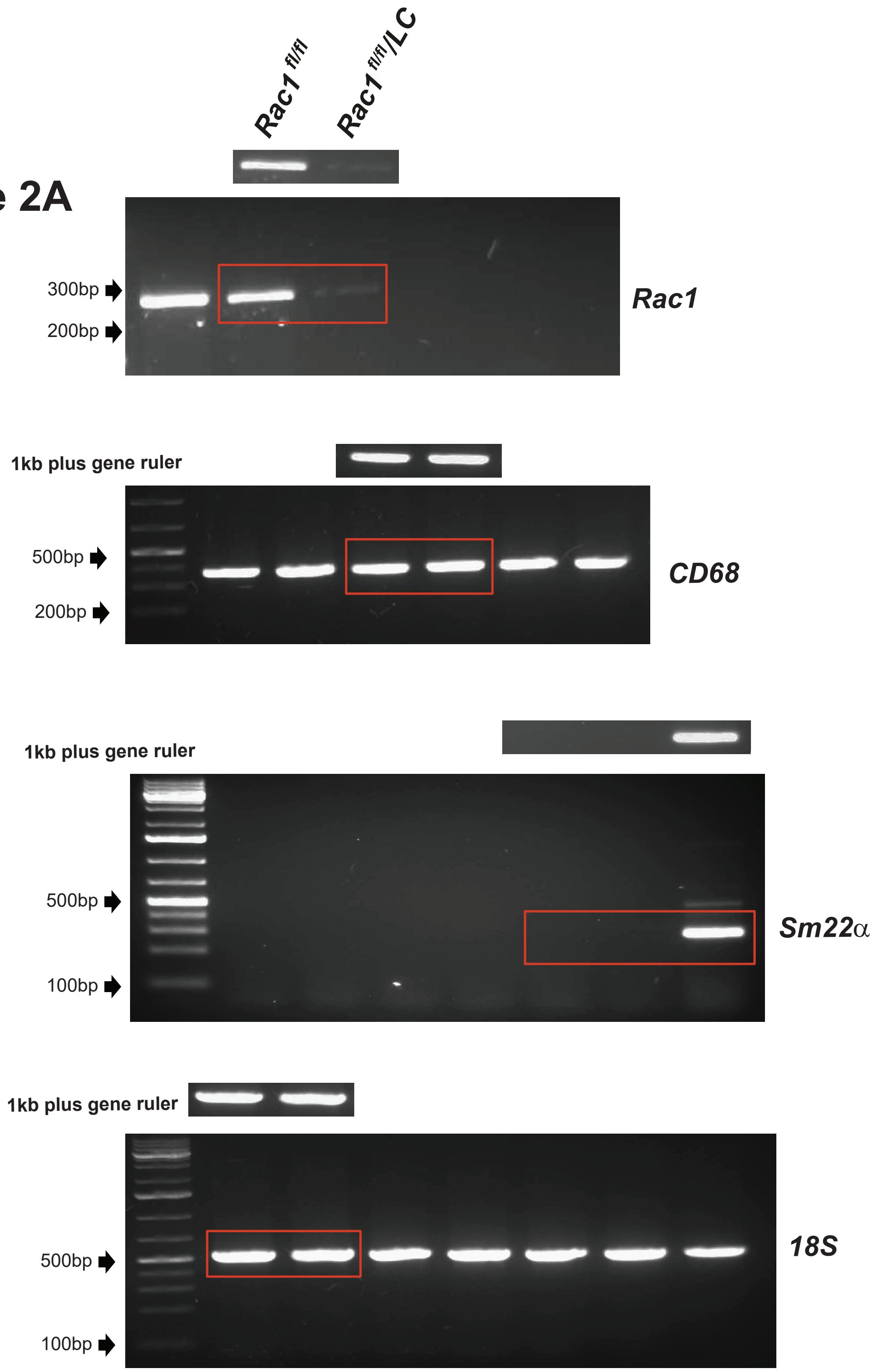

Figure 2B

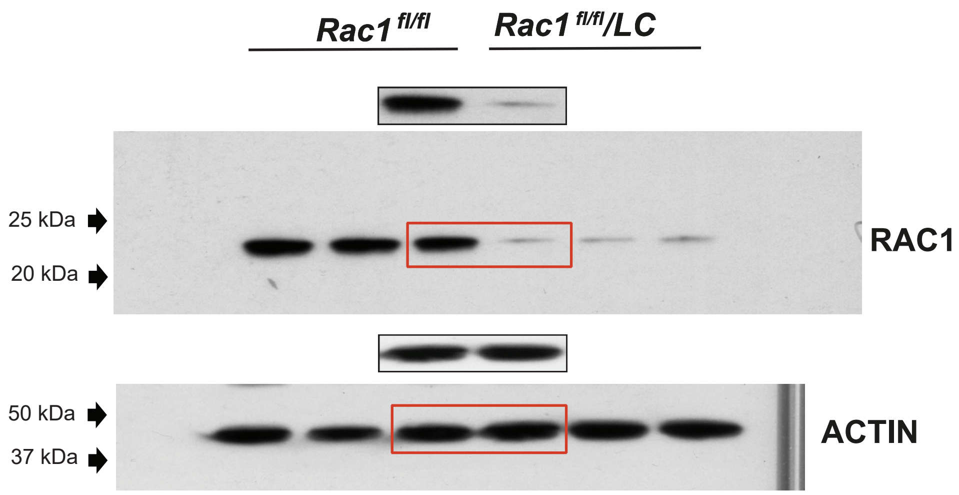

Figure 5B

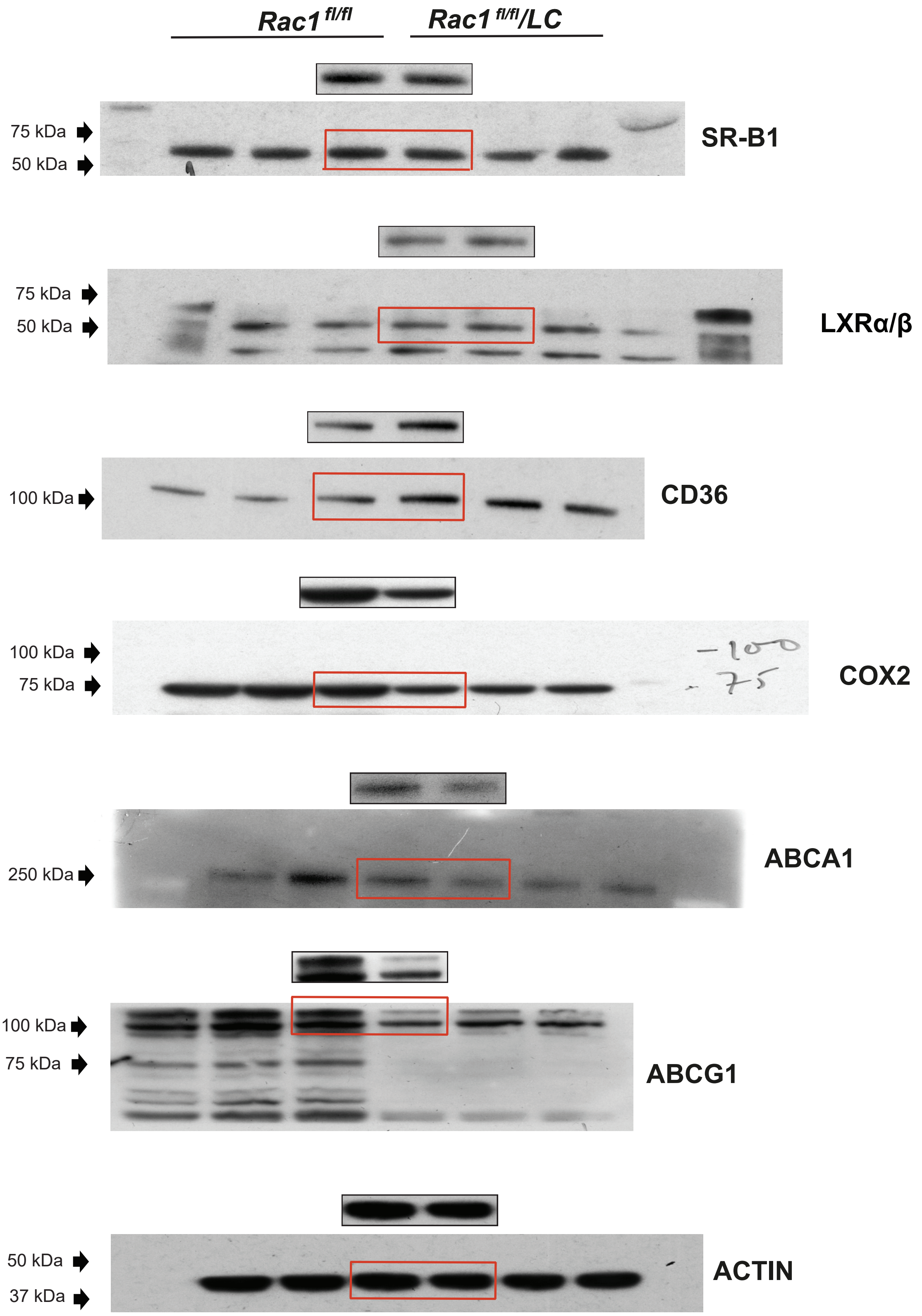

Rac1 fl/fl
Rac1 fl/fl/LC
75 kDa
50 kDa
SR-B1
75 kDa
50 kDa
LXRα/β
100 kDa
CD36
100 kDa
75 kDa
-100
75
COX2
250 kDa
ABCA1
100 kDa
75 kDa
ABCG1
50 kDa
37 kDa
ACTIN
