# Supplementary material for: Lack of RAC1 in macrophages protects against atherosclerosis
Source: PLoS One. 2020 Sep 17;15(9):e0239284. doi: 10.1371/journal.pone.0239284 (PMC7498073; doi:10.1371/journal.pone.0239284)
Supplement: Attachment — Submitted filename: Response to Reviewers.docx [file pone.0239284.s003.docx]

**Reviewer #1:**

1/ While the response letter suggests the authors included the naïve controls, this information is not included in the figures. This should be included in the figures of the paper. Also, I did not receive the “attached figure” mentioned.

“As requested, additional experimental groups without LPS have been included in experimental setting. We tested untreated BMMs for ELISA (Reviewer Figure 1) as well as for immunoblotting (Reviewer Figure 2), however, these assays did not result in significant differences. Please see the attached figure including these untreated BMMs along with LPS-treated BMMs to convince the Reviewer.”

We do apologize for any technical error that may have occurred during the submission procedure. We are now re-attaching these figures to the end of our Response letter for the Reviewer’s information.

ELISA analysis revealed no detectable levels of IL-6, IL-10, IL-12 or TNF-α in cultured BMMs without LPS (Reviewer Figure 1). Furthermore, immunoblotting showed that protein levels did not alter between the groups of BMMs (with or without RAC1) without stimulation with LPS (Reviewer Figure 2). Please notice that these figures will not be included for publication.

2/ The text should be copyedited by a professional to improve readability and correct mistakes. A few have been corrected but orthers remain. E.g. stimulated by LPS -> stimulated with LPS.

The manuscript has been linguistically reviewed by a native English-speaking scientist and her contribution has been acknowledged in the manuscript.

**Reviewer #2:**

1. The authors state that “it is known that RAC1-deficiency increases extension of filopodial formation; however, filopodial formation was reduced”. However, on the picture show it seems that filopodia are increased in the KO. Does this mean that the picture presented in Fig. 2C is not representative?. Moreover, the text added in the discussion does not clarify the observation to me.

It is known that RAC1-deficiency reduces filopodial formation in macrophages, but filopodial structures that are formed display increased extension (Wells CM, et al. J Cell Sci. 2004;117:1259–1268). In our study, we have not repeated these experiments; however, images represented in Figure 2C support that macrophages deficient for RAC1 display increased extension of filopodial formation. Please note that filopodial structures are longer, but fewer in number in RAC1-deficient macrophages compared to RAC1-expressing macrophages. This has been clearly stated in the Discussion (page 13, lines 13–16).

2. The authors state that they hopefully will perform more detailed analysis of plaque parameters in the future. However, given that they have paraffin embedded slides and HE stainings, I see no reason why it would not be possible to do a scoring of necrotic core on these sections and one additional Sirius red staining (which is relatively simple to perform).

We have now sectioned the remaining distal parts of aortic arches and stained them with Sirius red, as suggested. The number of red color pixels representing collagen composition, regardless of the extent of intimal thickening, have been quantified as percentages with an image analysis software. There was no difference in the percentage of collagen composition between the aortic sections from mice expressing or lacking RAC1 in macrophages. These data have been mentioned in the Results and presented as supplemental data (S3 Fig).

In Sirius red-stained aortic sections, we identified necrotic cores that we quantified as the percentage of intimal thickened areas, as suggested. In aortic sections that are lacking RAC1 in macrophages, the size of necrotic cores was significantly reduced. This data has been included as a new figure panel (Figure 3C). One should remember that the extent of intimal thickening is different between the groups (Figure 3A), but we presented data in percentages regardless of the magnitude of intimal thickening.

3. It remains unclear whether IL10 and IL12 were not measured in vivo or not presented. I would also argue to add the measurements of TNF in vivo to the manuscript and to discuss these.

As previously mentioned, we tried to measure in vivo serum levels of IL-10 and IL-12 but they were under detectable levels. These data have been mentioned in the Results (page 11, lines 20–21).

As suggested, we have included the data on the in vivo level of TNF-α between the experimental groups (new Figure 4B), although these levels did not reach statistical significance. Data presenting the in vitro levels of IL-10 and IL-12 between the cultured macrophages with or without RAC1 have been removed from Figure 4, as they did not yield any statistical differences. Nevertheless, these data have been mentioned in the Results (page 11, lines 18–21).
